# Analysis of Job-Related Demands and Resources in Ambulatory Youth Welfare Services: A Qualitative and Quantitative Approach

**DOI:** 10.3390/ijerph17082941

**Published:** 2020-04-24

**Authors:** Sylvie Vincent-Höper, Julia C. Lengen, Maren Kersten, Sabine Gregersen

**Affiliations:** 1 Department of Work and Organizational Psychology, Universität Hamburg, 20146 Hamburg, Germany; 2 Institute for Occupational and Maritime Medicine (ZfAM), University Clinic Hamburg-Eppendorf, 20459 Hamburg, Germany; j.lengen@uke.de; 3 Department of Occupational Medicine, Hazardous Substances and Public Health, Institution for Statutory Accident Insurance and Prevention in the Health and Welfare Services, 22089 Hamburg, Germany; maren.kersten@bgw-online.de (M.K.); sabine.gregersen@bgw-online.de (S.G.)

**Keywords:** social workers, ambulatory youth welfare, job demands, job resources, questionnaire, validation

## Abstract

In this study, we investigated health-relevant job characteristics of social workers in ambulatory youth welfare services, combining qualitative and quantitative methods. Based on a systematic literature review, expert workshops, and focus group discussions with 9 experts of the target group, we identified target group-specific job demands and job resources, which we compiled into a questionnaire using content-valid scales. The target group-specific survey tool comprises 9 scales for assessing job demands and 10 scales for assessing job resources. Analyses of data from 209 social workers demonstrated desirable psychometric properties and substantial correlations of the scales with coping behaviours and indicators of employee well-being. The scales for assessing job demands were negatively related to psychological well-being and job satisfaction and positively related to burnout and depressiveness. The scales for assessing job resources showed positive correlations with indicators of positive well-being and negative correlations with indicators of impaired well-being. Regression analyses revealed that job resources explained a higher amount of variance in the positive well-being indicators compared to job demands. The study identified a broad range of health-relevant job characteristics for social workers in ambulatory youth welfare. Applying the target group-specific survey tool allows organisations to derive suitable implications for the design of health promotion programs.

## 1. Introduction

In the light of demographic change and the associated shortage of skilled staff, social services organisations face the challenge of maintaining the health of well-qualified employees of all ages in the workplace over the long term [1]. However, this is a difficult endeavour. An analysis of data from health insurers and the German Social Accident Insurance (DGUV) showed that across all funds, mental disorders caused most sick leave days of social workers. About 21% of sick leave days were caused by mental disorders. Respiratory diseases were the second most common cause of sick leave (about 18%), followed by musculoskeletal diseases [2]. 

In general, the link between working conditions and mental and physical disorders has been well-documented [3,4,5,6], providing insights into the job demands and resources that should be addressed to enhance employee health. For social work, the picture is less clear. Compared to other occupational groups in the healthcare and social services sector, empirical studies examining job characteristics in social work that affect health are relatively scarce and fragmentary. In particular, the job demands and resources of social workers have not yet been investigated very systematically and the findings of the few studies on this issue are not easily comparable. One reason for this is the heterogeneity that exists within this occupational group, which delivers services such as assistance with parenting, child development advice, or social education support for families.

An important area of social work is youth welfare. For years, both the take-up of social services and the number of employees at youth welfare institutions has been on the rise [7]. Today, more than 260,000 people are employed in youth welfare in Germany, of whom a large proportion provide ambulatory youth support services [8]. Despite the importance of this work for society and the large number of employees, hardly any empirical data on the links between job characteristics and the mental health of workers in ambulatory youth welfare services exists. This lack of empirical findings may be due to the fact that there is no scientifically-based tool for assessing the health-relevant job characteristics of workers in ambulatory youth welfare services. This is unfortunate given that knowledge of the job demands and resources of workers in ambulatory youth welfare services is essential for an effective prevention and health promotion strategy aimed specifically at this target group [9].

To address this issue, the aim of this study is to identify health-relevant job characteristics of workers in ambulatory youth welfare services. Specifically, we aim to generate robust findings indicating which job characteristics have a particularly strong relationships with health for this specific occupational group. In the first step, we define job characteristics of workers in ambulatory youth welfare services which are relevant for the health of this target group using a qualitative approach. Second, we use the qualitative results to operationalise the job characteristics of workers in ambulatory youth welfare services with valid scales and investigate associations with different indicators of health using a quantitative survey. 

This study contributes to the existing literature in several ways. The structured, stepwise, and theory-based approach in exploring health-relevant job characteristics for workers in ambulatory youth welfare services contributes to a systematic analysis of job demands and resources of this specific target group. This systematic approach is particular in that it combines qualitative and quantitative elements. While the qualitative study serves to identify the target group’s health-relevant job characteristics, the results of the quantitative survey provide insights into the relevance of these specific job demands and resources for workers’ health. Finally, this study advances the understanding of which job characteristics are especially relevant for the development of effective health promotion programs for this specific target group. 

## 2. Theoretical Background

As theoretical framework for the development of the measure, we use the widely accepted Job-Demands-Resources-Model (JD-R-Model) [10]. The JD-R model divides job characteristics into demands and resources. According to the model, an accumulation of demands (e.g., time pressure, unfavourable environmental conditions) places a strain on physiological and mental health, leading to negative outcomes such as exhaustion or burnout [11]. By contrast, resources (e.g., autonomy, support, feedback) have a positive impact and result in higher engagement and better performance at work through a motivational process. Demands and resources also interact with one another, meaning that the available resources can soften the negative impact of the demands (ibid.). Additionally, the model assumes that high demands require workers to make more effort. The resulting response to demands—known as coping behaviour—can be very diverse [12].

To verify the relevance of the job characteristics to health, the focus is on mental health as a dependent variable. Well-being is a broad and multidimensional concept [13] that includes not only the absence of negative symptoms (e.g., strain and emotional exhaustion), but also involves positive states (e.g., happiness and work engagement). However, strongly related positive and negative indicators of well-being are assumed to represent two different dimensions instead of two poles on a continuum [14,15]. That is, low levels of negative symptoms are not necessarily associated with high levels of positive well-being. Therefore, we adopt a holistic view of employee well-being and assess both negative indicators (e.g., burnout) and positive indicators of employee well-being (e.g., job satisfaction). 

## 3. Methods

**Figure 1:**
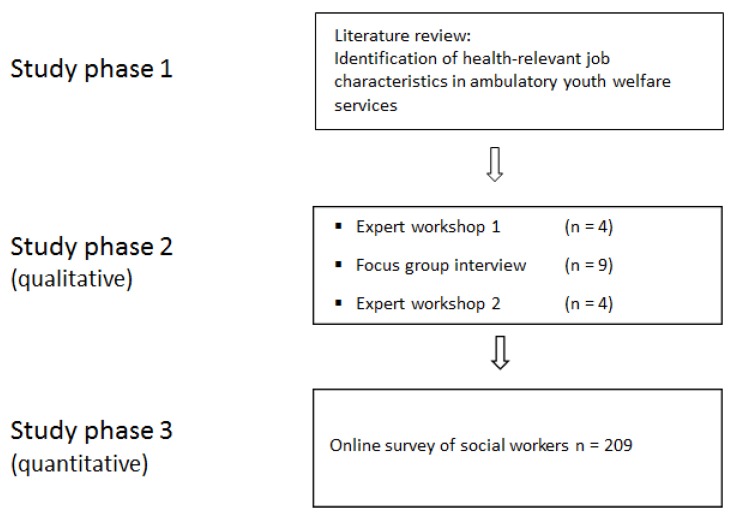
Identification and operationalisation of health-relevant job characteristics in ambulatory youth welfare services.

To identify the health-relevant job characteristics for workers in ambulatory youth welfare services, a systematic literature review was first conducted on the occupational and health situation in ambulatory youth welfare services. Based on the findings of the review, a group of experts in industrial psychology and a health scientist discussed relevant and appropriate scales for the quantitative assessment of the identified job characteristics [16]. Then, a focus group discussion was held with employees from ambulatory youth welfare services. They discussed which job characteristics have a bearing on their mental health and prioritised the job characteristics. The qualitative focus group data was analysed and presented to the experts again. In another workshop, they then selected the scales that were suitable for the quantitative assessment. Next, the questionnaire that was developed in the course of this iterative process was incorporated into a quantitative study using an online survey. The results of the quantitative survey were used to draw conclusions about the relevance of these job characteristics for workers’ health. Figure 1 shows the systematic approach to identifying health-relevant job characteristics and their operationalisation.

### 3.1. Systematic Literature Review: the Occupational and Health Situation in Ambulatory Youth Welfare Services

The systematic literature review by Lengen et al. (in prep.) [17] summarises the current state of scientific knowledge concerning the associations between job characteristics and workers’ mental health in ambulatory youth welfare services. Ten studies were assessed in this review. The results of the systematic literature review provide the basis for the next steps and are summarised below. Based on the current state of scientific knowledge, it was possible to identify a number of demands and resources that are relevant for the health of workers in ambulatory youth welfare services. 

The demands shown as significant for mental health were role conflicts [18,19], workload [19,20,21,22,23], social demands (clients), and emotional demands [18,19]. Conflicts with managers represented a further demand [21]. Qualitative studies list issues such as insufficient human and financial resources [7], control and bureaucracy from policy-makers and authorities [7,19], and insufficient time to interact with colleagues as having a bearing on health [21]. To date, the characteristics of unpredictability and high responsibility have also only been qualitatively investigated and identified as relevant factors [19]. 

Among the resources, job autonomy, shared values [23,24], and role clarity [20] showed the strongest correlations with the health indicators. Furthermore, the sense of being appreciated and participation were highlighted as significant resources [24]. Regular interaction with colleagues was also cited in several qualitative studies as an important resource [21,22]. The existing studies do not unanimously identify feedback and social support as relevant resources [20,22,25] and there is no clear evidence to support the relevance of meaningful work for health [20,25].

### 3.2. Qualitative Study

To establish health-relevant job characteristics for the specific target group of workers in ambulatory youth welfare services, workshops were held with four experts from healthcare and research. A focus group discussion was conducted with nine professionals from the field of ambulatory youth welfare services. The aim of the workshops and focus group discussion was to identify and operationalise relevant job characteristics for the subsequent quantitative assessment.

#### 3.2.1. Expert Workshop 1

The sample for the two expert workshops comprised three industrial and organisational psychologists—one of them from the University of Hamburg—and a health scientist from the University Medical Centre Hamburg-Eppendorf. All of the participants were female. Their ages ranged from 35 to 57. Their length of employment in this area was between 8 and 25 years. At the first expert workshop, the results of the systematic literature review were presented to the experts. They then discussed the relevance of the job characteristics for health. Scales from validated instruments were selected for the job characteristics that were deemed relevant by the experts. The experts considered several scales for a number of the job characteristics. Additional information was needed from the focus group before a final decision could be made. 

#### 3.2.2. Focus Group Discussion

The sample for the focus group was selected to be as heterogeneous as possible with respect to age, gender, length of service, qualifications, and field of work [26]. The focus group discussion involved nine workers from various areas of ambulatory youth welfare. Seven of the interviewees were women and age ranged between 29 and 54 years (mean age: 39 years). The duration of their actual position in youth welfare services ranged between one and 17 years. They worked in many different areas (e.g., ambulatory assistance with parenting, child development advice, social education support for families, non-specific child and youth work). The discussion process was structured using a guideline that served as guidance for the moderator and ensured that all the relevant aspects were covered during a focus group meeting [27]. The focus group defined relevant job characteristics from the perspective of the target group. Subsequently, each of the nine members of the focus group was assigned ten points to score the health-related relevance of the defined job characteristics. The participants were allowed to score a maximum of two points per construct, resulting in a frequency ranking of the job characteristics. 

**Figure 2:**
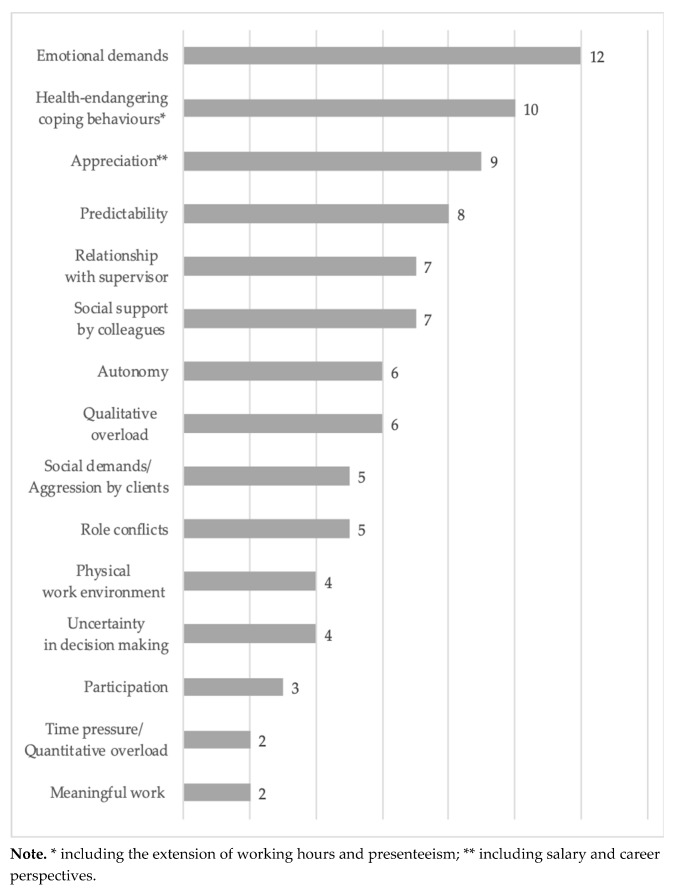
Prioritisation of issues at the focus group discussion.

The group discussion material had to undergo a standardised content analysis to ensure a valid evaluation [28]. The content was evaluated using the Mayring method of qualitative content analysis [29]. The result was a prioritisation of the constructs based on relevance for the target group, ranked using the point score awarded by the participants. The resulting prioritisation can be seen in Figure 2. Among the demands, emotional demands scored particularly highly. A number of resources proved similarly relevant (e.g., support, predictability). In addition to the key job characteristics, coping behaviours demonstrated in response to a demand (e.g., time pressure) were also cited, including health-endangering coping behaviours such as extension of working hours and presenteeism. Conceptually speaking, these are not job characteristics; they are coping behaviours which serve to explain the link between demands and health. Due to their high point score, these personal coping behaviours were included in the survey as an additional criterion variable, the assumption being that they correlate positively with demands (especially quantitative or qualitative demands).

#### 3.2.3. Expert Workshop 2

The subject of the second expert workshop was the composition and adjustment of the relevant scales within the framework of the JD-R model, incorporating the results of the focus group discussion. Following the JD-R model, the health-relevant job characteristics were categorised as demands and resources. 

The result of the qualitative investigation was a questionnaire containing nine demands, ten resources, and six criterion variables (two coping behaviours and four health indicators). Next, this questionnaire was checked in a quantitative study.

Based on the theoretical considerations of the JD-R model, the following hypotheses were formulated:

**H1:**  *The demands correlate positively with the health-endangering coping behaviours*.

**H2:**  *The demands correlate negatively with the indicators of well-being and positively with the indicators of impaired well-being*.

**H3:**  *The resources correlate positively with the indicators of well-being and negatively with the indicators of impaired well-being*.

**H4:**  *The demands explain greater variance in the indicators of impaired well-being than the resources, while the resources explain greater variance in the well-being indicators than the demands*.

### 3.3. Measures

The questionnaire is composed of different sociodemographic variables, a variety of job demands and job resources, coping behaviours, and indicators of employee well-being. Table 1 shows the scales selected on the basis of the qualitative study. 

#### Sociodemographic Variables

Standard sociodemographic details are collected such as field of work, occupation [19], professional experience [20], professional qualifications [18,24], managerial responsibilities [30], time limits on employment contracts [19,24], contractual and actual working hours [31,32], gender, and age [33]. 

### 3.4. Quantitative Study

#### Sample and Procedure

The target group for this survey comprises workers who deliver advice, assistance, or support to young people and their families through ambulatory services. To recruit a suitable sample for the quantitative validation study, associations that represent workers in ambulatory youth welfare services were contacted and asked to publicise the survey link on their websites, in newsletters, via corresponding social media groups, or via other types of networking. This acquisition approach yielded a convenient sample comprising employees of many different organisations. The sociodemographic data captured included the respondents’ occupation to ensure that no data was collected from people who do not belong to the target group. In programming the questionnaire, we defined all items as mandatory, meaning that the respondent had to fill out all items to complete the questionnaire. Therefore, we have no missing values and included only complete questionnaires in the analyses.

### 3.5. Data Analysis

#### 3.5.1. Verifying Reliability

A measure of internal consistency (Cronbach’s alpha) was used to determine the reliability of the scales. The minimum requirements for reliability are between 0.65 and 0.7. Values between 0.7 and 0.8 are respectable; 0.8 to 0.9 is good. If the score exceeds 0.9, shortening the scale should be considered [45]. Scales meeting at least the minimum requirements according to Everitt & Skrondal (2010) [45] were accepted for the quantitative assessment. As Cronbach’s alpha is not meaningful for scales with just two items, the Spearman-Brown correlation coefficient was provided as a measure of reliability for these scales. This requires strong correlations with values of at least *r* > 0.6 [46]. 

#### 3.5.2. Verifying Criterion Validity

To verify the criterion validity, correlations were calculated between the job characteristics, coping behaviour, and mental health [46]. These correlations can be used to draw conclusions about the health relevance of the job characteristics. As regards field studies which examine links between job characteristics and workers’ physical and mental health, it must be noted that the maximum correlations are usually around r = 0.20 and r = 0.30 [47]. Therefore, correlations of r = 0.25 or more are viewed as relevant because they make a significant contribution towards explaining the state of health associated with work. The explained variance in the well-being indicators resulting from the group of demands and the group of resources was also analysed using multiple regressions.

## 4. Results

### 4.1. Descriptive Statistics 

The sample comprised 209 workers from ambulatory youth welfare services. The majority of the study participants were female (70%). The average participant age was 44.2 years (SD = 11.31) and the average length of professional experience was 14.9 years (SD = 10.2). Their actual number of working hours was 35.49 (SD = 9.14). Further, 30% of the respondents had a managerial function. The majority worked in ambulatory youth welfare (87.1%), 8.6% worked in ambulatory child and youth services, and 3.8% were involved in the provision of quasi-residential youth welfare services. Almost half were qualified social workers or social education workers (49.8%) and 20.6% held a different education qualification. Additionally, 7.7% had trained as early years practitioners, 2.9% had another vocational qualification in education, 18.7% held a different vocational qualification, and 88.5% had a permanent employment contract. 

### 4.2. Reliability and Correlation Analyses

Table 2 shows the correlations between the job characteristics with the coping behaviours extension of working hours and presenteeism and both the positive and negative indicators of well-being. There are strong positive correlations between coping behaviours and demands. The correlations with resources are considerably less pronounced. Hypothesis 1 can therefore be confirmed. The correlation analyses with the indicators of well-being show that the demands correlate negatively with the positive health indicators (job satisfaction and well-being) and positively with the negative health indicators (mental exhaustion, depressiveness). As expected, the resources correlate positively with the positive indicators and negatively with the negative indicators of mental health. Almost all of the correlations between the job characteristics and the health indicators can be termed significant. The scale “aggression by clients” alone exhibits less pronounced correlations below the cut-off value of r ≥ 0.25. All of the other job characteristics exhibit correlations of r ≥ 0.25 with at least one health indicator. Thus, Hypotheses 2 and 3 can be confirmed. Overall, just 12% of the correlations are r ≤ 0.20, 43% of the correlations range between r = 20 and r. = 0.29, 28% of the correlations range between r = 30 and r. = 0.39, and even 17% of the correlations are r ≥ 0.40. The strongest correlations among the demands were displayed by role conflicts (r = −0.51 to r = 0.37) and qualitative overload (r = −0.45 to r = 0.33), followed by uncertainty in decision making (r = −0.32 to r = 0.38), emotional demands (r = −0.35 to r = 0.36), physical work environments (r = −0.40 to r = 0.27), time pressure (r = −0.31 to r = 0.26), and hiding emotions (r = −0.25 to r = 0.25). Among the resources, a host of job characteristics consistently returned strong correlations with the health indicators, such as appreciation (r = −0.32 to r = 0.59), predictability (r = −0.29 to r = 0.52), autonomy (r = −0.33 to r = 0.42), feedback and recognition from the supervisor (r = −0.29 to r = 0.44), social exchange (r = −0.26 to r = 0.44), meaning of work (r = −0.27 to r = 0.40), and social support by colleagues (r = −0.27 to r = 0.35). 

### 4.3. Multiple Regression Analyses

To verify the relevance of the individual groups of job characteristics (demands and resources) for the various health indicators, multiple regression analyses were used to check the explained variance of the respective groups of job characteristics for each health indicator. First, the sociodemographic variables of gender, age, and working hours per week were included in the regression model. Next, the group of demands—respectively, the group of resources—were incorporated. The results show that the demands explain a variance of 41% in the job satisfaction variable and 19% in the well-being variable over and above the control variables. In the impairments to well-being, the demands explain 18% in mental exhaustion and 20% in depressiveness. The resources explain a variance of 70% in job satisfaction and 23% in well-being. As regards the impairments to well-being, the resources explain 18% in mental exhaustion and 22% in depressiveness. Overall, the resources explain far greater variance in the positive health indicators. Contrary to the assumption, the resources and the demands each explain a similar amount of variance in the impairments to well-being. For this reason, hypothesis 4 can only be confirmed in part. 

## 5. Discussion

### 5.1. Summary

This study’s objective was to identify health-relevant job characteristics for workers in ambulatory youth welfare services and to investigate links to mental health via a quantitative survey. Using these results, it is possible to generate robust findings concerning which job characteristics are particularly relevant for this sample’s health. 

For this purpose, a content-validated questionnaire was developed, which was tailored explicitly to the working conditions of this target group. This work was based on the JD-R model and a systematic literature review and was supported by expert workshops and the participative, qualitative element of a focus group discussion with workers from ambulatory youth welfare services. The questionnaire scales were verified for reliability and criterion validity by means of a subsequent quantitative study that used an online survey. 

Demands and resources were covered in roughly equal measure. The scales exhibit good psychometric properties. The correlation analyses can be viewed as confirmation of the criterion validity and thus, also of the relevance of the selected demands and resources for health of this target group. The scale “violence by clients” was the only one with lower correlations. As this issue was considered to be very important by the experts, the inclusion of this aspect in future studies, possibly using a different scale, is recommended. Alternatively, the scale “violence by clients” could be adjusted in line with a survey tool for clinicians [48]. 

The result that the demands correlate much more strongly with health-endangering coping behaviours is plausible and conforms to theory when one considers that this coping behaviour is exhibited in response to demands (e.g., time pressure; [41]).

Overall, the resources explain far greater variance in the positive health indicators, which conforms to the theory behind the JD-R model [10]. The fact that the resources explain a similar amount of variance in the impairments to well-being contradicts a fundamental assumption of the JD-R model. This finding indicates that work-related resources play a particularly important role for the mental health of employees in this specific target group. 

In summary, it is fair to say that the correlations between job characteristics and health indicators are very significant [47], meaning that the job characteristics identified have a high health relevance for the target group of workers in ambulatory youth welfare services. Among the demands, role conflicts and qualitative overload proved particularly significant with regard to mental health. Of the resources, a number of resources seem to play an important role in mental health (such as appreciation, predictability, autonomy, feedback and recognition from managers, social exchange and support by colleagues, and meaning of work). 

Previous studies examining job characteristics in ambulatory youth welfare services that affect health are scarce and fragmentary [17]. Thus, job demands and resources in ambulatory youth welfare services have not yet been investigated systematically. While the findings of this study are consistent with findings of previous studies on the relevance of specific job characteristics (e.g., role conflicts, appreciation) for mental health [18,19,23], we add to the literature by identifying a broad range of health-relevant job demands and job resources. Thus, this study contributes to a systematic analysis of job demands and resources of this specific target group and paves the way for the development of effective health promotion programs in ambulatory youth welfare services. 

### 5.2. Limitations

Although the construct of interest can be identified more reliably with three or more items than with two [49], several short scales consisting of just two items were used in this quantitative survey to capture the job characteristics. The reason for this is that it enables coverage of the breadth of content within the constructs recorded in the qualitative study, whilst at the same time preventing fatigue among respondents by asking them to complete an overly lengthy questionnaire. 

A methodical limitation that could distort the results is the single-source bias that arises because both the job characteristics and the criterion variables were rated by the same person. The common method variance gives rise to the risk that “correlations with variables measured using the same method may be inflated by common method variance” [50]. 

A convenient sample was used for the survey. A larger sample would be desirable for future studies. Furthermore, it should be noted that data was cross-sectional. A longitudinal study would be needed to verify the relevance of the job characteristics for the employees’ health in the long-term.

It remains unclear to what extent the findings are specific for employees in ambulatory youth welfare services and whether they can be generalised to other occupations in social work. Future studies should investigate whether this target group-specific questionnaire with the demands and resources can also be used for other fields of social work. 

### 5.3. Implications for Research

In future research, the validated questionnaire can be used to assess the demands and resources in ambulatory youth welfare services to examine their potential impact on mental health. This could fill a gap in industrial psychology research relating to the group of workers in ambulatory youth welfare services. By using the questionnaire, the specific demands and resources which apply to this occupational group can be investigated systematically at the organisational level. Workplace health promotion measures can be determined using targeted prevention programmes and their effectiveness can be evaluated systematically using the tool. 

As the previous knowledge of job characteristics was derived solely from cross-sectional studies [51], the prime recommendation is to use the tool developed in this research for a longitudinal study to take developments over time into account and be able to investigate causal links between the job characteristics and health indicators. 

Furthermore, future studies can investigate the interplay between demands and resources and uncover possible buffer effects that resources may have on the negative health impact of the demands, based on the JD-R model [10]. 

### 5.4. Implications for Practice

The findings of this study offer considerable potential for use by ambulatory youth welfare institutions to establish risks to mental health when they perform risk assessments. With the aid of the survey results, it is possible to list specific demands (e.g., role conflicts and qualitative overload) and resources (e.g., appreciation, predictability, autonomy, feedback and recognition from managers, social exchange and support by colleagues, and meaningfulness) that are important for mental health. When deriving measures, it would be prudent to select options that address both the situation in the workplace and employees’ behaviour. According to Bamberg & Busch [52], stress management programmes of this kind also have the most lasting effect. Special attention should be paid to the link between demands and coping behaviours. 

## 6. Conclusions

This study’s objective was to identify the health-relevant job characteristics for workers in ambulatory youth welfare services and to investigate them using a validated, target group-specific questionnaire. Combining qualitative and quantitative methods within a systematic, iterative process allowed health-relevant job characteristics for this specific target group to be recorded in a valid fashion. The survey tool developed as part of this research has both scientific and practical uses and makes a promising contribution towards preventive and prospective job design and health promotion. The findings gained with the aid of this tool are a key prerequisite for effective target group-oriented prevention and health promotion. This research can now be built upon and future studies can investigate the association between job characteristics and mental health in greater depth. This in turn will pave the way for intervention recommendations aimed specifically at this target group. 

## Figures and Tables

**Table 1:** Scales of the questionnaire.

Scale	Number of Items	Range	Sample Item	Reference
**A. Demands**				
Emotional demands	3	1–5	How often is your work highly demanding?	COPSOQ [34] (Nübling et al. 2017); slightly adapted
Hiding emotions	3	1–5	How often does your work require that you hide your feelings?	COPSOQ [34] (Nübling et al. 2017); slightly adapted ISAK-K [35] (Keller et al. 2013)
Quantitative overload	3	1–5	How often are you under time pressure?	COPSOQ [34] (Nübling et al. 2017) ISAK-K [35] (Keller et al. 2013)
Uncertainty in decision making	3	1–5	How often do you have to make decisions without sufficient information?	ISAK-K [35] (Keller et al. 2013) developed by the authors (1)
Qualitative overload	4	1–5	Sometimes I have to do things for which I am not sufficiently trained.	SALSA [36] (Rimann &Udris 1997) developed by the authors (1)
Social demands by clients	4	1–5	How often do clients have too high expectations on you?	ISAK-K [35] (Keller et al. 2013) developed by the authors (2)
Aggression by clients	2		Did you experience physical aggression by clients in the last 12 months?	Schablon et al. 2018 [37]
Role conflicts	4	1–5	Are contradictory demands placed on you at work?	COPSOQ [34] (Nübling et al. 2017) developed by the authors (2)
Physical work environment	5	1–5	Are you affected at work by the following things? -noise	SALSA [36] (Rimann &Udris 1997)
**B. Resources**				
Autonomy	3	1–5	The job allows me to plan how I do my work.	WDQ [38] Autonomy (Stegmann et al. 2010)
Participation	4	1–5	If someone has a good idea, it is possible to put it into practice in this company.	SALSA [36] (Rimann & Udris 1997) and developed by the authors (1)
Predictability	2	1–5	At your place of work, are you informed well in advance concerning, for example, important decisions, changes, or plans for the future?	COPSOQ [34] (Nübling et al. 2017)
Appreciation	3	1–5	Personal engagement and willingness to perform pays off in this organisation.	DiGa [39] (Ducki 2000) COPSOQ [34] (Nübling et al. 2017)
Meaning of work	2	1–5	Is your work meaningful?	COPSOQ [34] (Nübling et al. 2017)
Feedback/ recognition from the supervisor	3	1–5	My supervisor lets me know how well I do my work.	GEFA [40] (Vincent-Höper & Stein 2019)
Fairness/integrity from the supervisor	3	1–5	My supervisor makes sure that the work is fairly distributed among the employees.	GEFA [40] (Vincent-Höper & Stein 2019)
Social support from the supervisor	3	1–5	How much can you rely on your supervisor if problems occur at work.	SALSA [36] (Rimann & Udris 1997)
Social support from colleagues	3	1–5	How much can you rely on your colleagues if problems occur at work.	SALSA [36] (Rimann & Udris 1997)
Social exchange in teams	2	1–5	I have the opportunity to meet with other colleagues in my work.	WDQ [38] (Stegmann et al. 2010) slightly adapted and developed by the authors (1)
**C. Coping**				
Extension of working hours	3	1–5	How often did you make yourself available for your supervisor, colleagues, or clients during leisure time in the last three months?	Krause et al. 2014 [41]
Presenteeism	2	1–5	How often did you work despite being sick in the last three months?	Krause et al. 2014 [41]
**D. Indicators of mental health**				
Job satisfaction	1	1–5	Regarding your work in general. How pleased are you with your job as a whole, everything taken into consideration?	COPSOQ [34] (Nübling et al. 2017)
Well-being	5	1–6	In the last two weeks, I have felt cheerful and in good spirits.	WHO 5 [42]
Depressiveness	8	1–7	I have sad moods.	Mohr & Müller 2014 [43]
Personal Burnout	6	1–5	How often do you feel tired?	CBI [44] (Kristensen et al. 2005)

**Table 2:** Scale values and correlation coefficients.

	M	SD	Cronbachs’ Alpha/r_it_	Extension of Working Hours	Presenteeism	Job Satisfaction	Well-Being	Depressiveness	Personal Burnout
Emotional demands	3.16	0.60	0.72	0.18 **	0.40 ***	−0.32 ***	−0.35 ***	0.23 **	0.36 ***
Hiding emotions	3.21	0.79	0.74	0.11	0.12	−0.25 ***	−0.24 ***	0.21 **	0.25 ***
Quantitative overload	3.34	0.80	0.84	0.37 ***	0.31 ***	−0.31 ***	−0.30 ***	0.24 ***	0.26 ***
Uncertainty in decision making	2.88	0.68	0.67	0.33 ***	0.27 ***	−0.32 ***	−0.23 **	0.38 ***	0.27 ***
Qualitative overload	2.84	0.67	0.68	0.28 ***	0.22 **	−0.45 ***	−0.35 ***	0.33 ***	0.33 ***
Social demands (clients)	3.14	0.61	0.71	0.16 *	0.27 ***	−0.16 *	−0.22 **	0.23 **	0.25 ***
Aggression by clients	2.02	0.95	0.63	0.21 **	0.15 *	−0.16 *	−0.09	0.14 *	0.07
Role conflicts	2.64	0.72	0.81	0.25 ***	0.37 ***	−0.51 ***	−0.40 ***	0.34 ***	0.37 ***
Physical work environment	2.48	0.75	0.74	0.20 **	0.25 ***	−0.40 ***	−0.24 ***	0.17 *	0.27 ***
Autonomy	4.03	0.66	0.75	−0.16 *	−0.26 ***	0.42 ***	0.31 ***	−0.27 ***	−0.33 ***
Participation	3.10	0.97	0.86	0.02	−0.10	0.52 ***	0.26 ***	−0.21 **	−0.19 **
Predictability	3.65	1.04	0.68	−0.09	−0.20 **	0.52 ***	0.33 ***	−0.29 ***	−0.27 ***
Appreciation	2.84	0.83	0.76	−0.08	−0.15 *	0.59 ***	0.35 ***	−0.32 ***	−0.31 ***
Meaning of work	4.39	0.69	0.72	−0.01	−0.15 *	0.40 ***	0.34 ***	−0.27 ***	−0.24 ***
Feedback/recognition (supervisor)	3.09	1.12	0.93	−0.03	−0.09	0.44 ***	0.27 ***	−0.26 ***	−0.29 ***
Fairness/integrity (supervisor)	3.57	1.01	0.87	−0.07	−0.11	0.41 ***	0.21 **	−0.17 *	−0.22 **
Social support (supervisor)	3.85	1.03	0.91	−0.02	−0.08	0.47 ***	0.19 **	−0.13 *	−0.22 **
Social support (colleagues)	4.16	0.77	0.88	−0.07	−0.08	0.35 ***	0.30 ***	−0.27 ***	−0.26 ***
Social exchange (teams)	3.83	0.97	0.67	−0.21 **	−0.16 *	0.44 ***	0.27 ***	−0.25 ***	−0.26 ***

Note. * *p* < 0.05 ** *p* < 0.01 *** *p* < 0.001.
